# A new species of *Isoperla* (Insecta, Plecoptera) from the Karawanken, with considerations on the Southern Limestone Alps as centers of endemism

**DOI:** 10.3897/zookeys.448.8509

**Published:** 2014-10-20

**Authors:** Wolfram Graf, Martin Konar, Dávid Murányi, Kirill Márk Orci, Simon Vitecek

**Affiliations:** 1 Institute of Hydrobiology and Aquatic Ecosystem Management, University of Natural Resources and Applied Life Sciences, Max Emanuel-Strasse 17, A-1180 Vienna, Austria; 2 Amt der Kärntner Landesregierung, Abteilung 8 (Kompetenzzentrum Umwelt, Wasser und Naturschutz), Kärntner Institut für Seenforschung, 9020 Klagenfurt am Wörthersee, Kirchengasse 43, Austria; 3 Department of Zoology, Hungarian Natural History Museum, Baross u. 13, H-1088 Budapest, Hungary; 4 MTA-ELTE-MTM Ecology Research Group, Pázmány Péter s. 1/C, H-1117 Budapest, Hungary; 5 Department of Limnology & Bio-Oceanography, Faculty of Life Sciences, University of Vienna, Althanstrasse 14, A-1090 Vienna, Austria

**Keywords:** *Isoperla*, new species, endemism, Austria, Slovenia, Southern Alps

## Abstract

A new species of the genus *Isoperla* (Plecoptera, Perlodidae), belonging to the *oxylepis* species-group is described, and the male mating call is characterized. Its range falls within a small region of the Southern Limestone Alps which is well known to be one endemism-centre of aquatic insects.

## Introduction

The genus *Isoperla* consists of about 150 species (DeWalt et al. 2011, Baumann and Lee 2009, Murányi 2011, Szczytko and Stewart 1979, Zwick and Surenkhorloo 2005) and covers the Holarctic and Oriental regions. In Europe 56 species are known so far (Graf et al. 2009, Murányi 2011), of which ten occur in Austria (Graf 2010). *Isoperla* is a morphologically difficult genus, especially the *grammatica* and *tripartita* species groups that both exhibit high variability, and requires further resolution. A synthesis of zoogeographical, morphological, molecular, and possibly behavioural data will be required to get full knowledge on the diversity of this highly interesting genus.

Recently a series of specimens were collected from the Karawanken Alps in southern Austria and the nearby Kamnik Alps in northern Slovenia deviating from all hitherto known species. In this paper we provide morphological descriptions of males, females and the larva. Additionally we illustrate drumming signals of one male.

## Material and methods

Adult specimens were collected using sweep nets, larvae were collected by handpicking from cobbles (mesolithal), the dominant substrate type. Collected specimens were stored in 70% ethanol. Morphological characteristics of male terminalia were examined in KOH-treated, cleared specimens. Comparative material from the authors’ collections enabled the identification of the new species.

Vibratory signal recordings were made using a small, dynamic speaker (SAL YD78) as a vibration transducer. The speaker was connected to the microphone input socket of a solid state, digital recorder (Zoom H4n). The examined specimen was placed on the diaphragm of the speaker. To prevent the specimen from escaping the speaker was covered by a sheet of hobby glass. During the recordings ambient air temperature was measured using a P 300W thermometer. Vibration recordings were analysed and oscillograms produced using the software Adobe Audition 1.5 (Adobe Systems Incorporated, San Jose, California, USA). Drumming signal terminology follows Stewart and Sandberg (2006) and Murányi et al. (2014).

## Results

### Isoperla claudiae

Taxon classificationAnimaliaPlecopteraPerlodidae

Graf & Konar sp. n.

http://zoobank.org/50F79ECE-AD68-4DD1-BD7F-643D25205189

Figs 1 , 2 , 3

#### Type material.

Holotype: 1 male, Austria, Carinthia, Dolintschitschach brook south-east of Feistritz ob Bleiburg (46°32'6"N, 14°45'52"E), 600m a.s.l., 30.5.2014, leg. W. Graf; Paratypes: 3 males, 2 females, same place, date and collector. The holotype is deposited at the Linzer Landesmuseum, Linz, Austria, paratypes are stored in the first author's collection.

Other material. 1 male (drumming call examined), 1 female (HNHM: PLP4333), Slovenia, Upper Carniola, Kamnik municipality, Kamnik Alps, small forest brook S of Podvolovljek Pass (46°16.250'N 14°41.325'E), 980m a.s.l., 09.07.2013, leg. D. Murányi, I. Sivec.

#### Type locality.

Austria, Carinthia, Feistriz ob Bleiburg, Dolintschitschach brook.

#### Etymology.

The species is named in honour of the second author’s wife Claudia.

#### Diagnosis.

An *Isoperla* exhibiting the following combination of characters: (1) a small medial penial armature in the form of an equilateral triangle, lacking lateral penial armatures; (2) yellow head and pronotum with a small horseshoe-like brown marking connecting the occelli.

#### Description.

**Figure 1.**
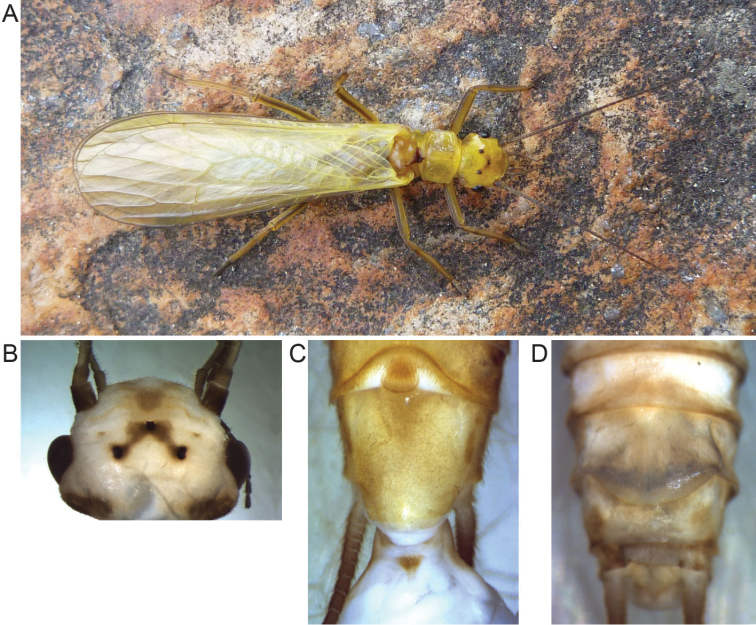
*Isoperla claudiae* sp. n. **A** habitus **B** colouration of the head of *Isoperla claudiae* **C** ventral view of the male abdomen with extruded penis **D** ventral view of the female abdomen.

Medium-sized species, macropterous. Body length: holotype 10.5 mm, allotypes 11–12 mm; forewing length: holotype 12 mm, paratypes 12–14 mm. Primary colouration yellow, head and pronotum mostly yellow with dark brown markings; pilosity short. Primary colouration of the head yellow, with a dark horseshoe-like brown patch connecting the three ocelli (Fig. 1A, B). Occiput with indistinct rugosities but with brown patches laterally. Eyes normal sized. Scape brown, pedicel and the following antennomeres brown; palpi greyish to light brown. Pronotum yellow with a delicate brownish marking at the posterior margin, trapezoidal, edges angled; rugosities hardly visible and yellow. Anterior part of the mesonotum yellow, remaining portions brown; metanotum medially dark brown, laterally and anterior of the insertion of wings whitish. Wings yellowish, particularly the anterior half; venation mostly whitish to yellow, costa and apical part of radii brown. Ventral surface of thorax pale, meso- and metabasisternum inconspicous, furcasternites and furcal pits pale. Femora brown dorsally and yellow ventrally. Tibiae brownish dorsally, pale ventrally; tarsi brown.

Male abdomen (Fig. 1C): 1^st^ to 7^th^ tergite dorsally brown (with some tiny pale spots) with increasing laterally whitish areas towards the apex, 8^th^ to 10^th^ tergite mostly yellowish with small brown medial patches and medially interrupted anterior stripes up to T9, T10 pale without markings. Laterally and ventrally all segments whitish to yellow, lacking dark markings. Pilosity on segment posterior ends short and inconspicuous. Ventral lobe of sternite VIII yellow, slightly longer than wide, its posterior margin strongly convex with long marginal pilosity. Sternite IX yellowish. Paraprocts brown, regularly curved in caudal with with blunt tips; cerci light brown, apically dark brown.

**Figure 2.**
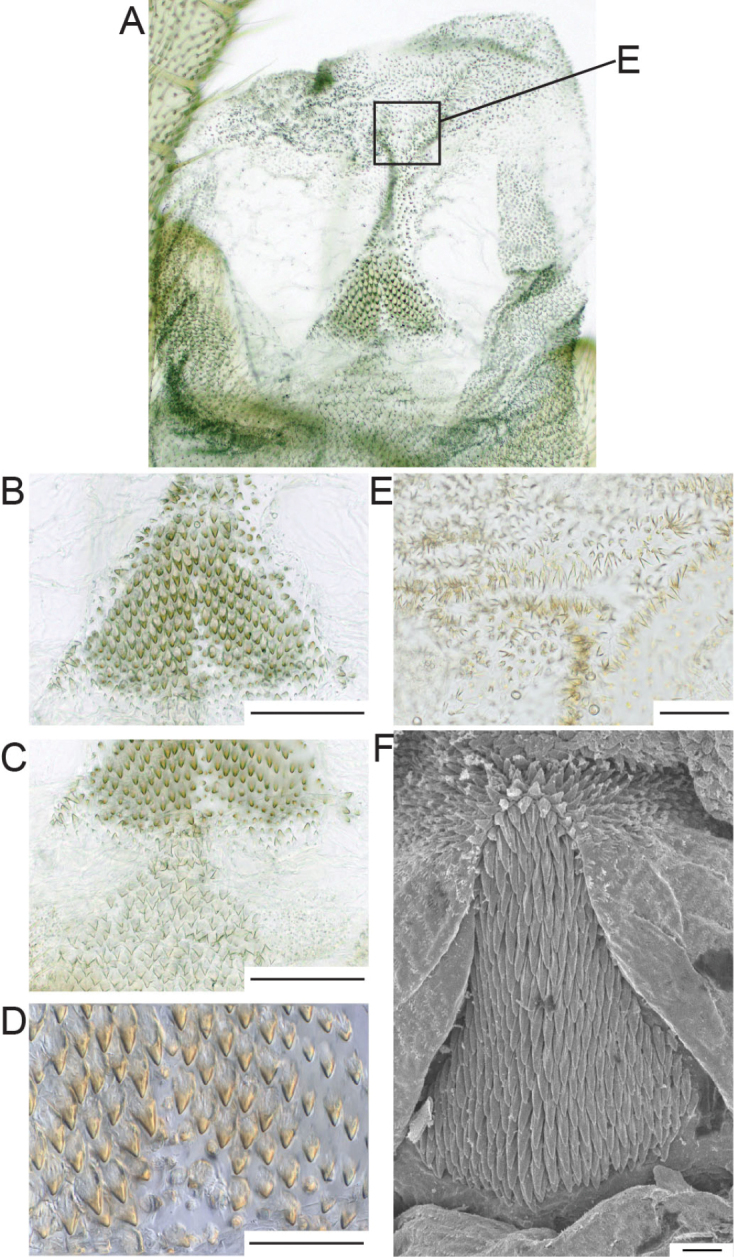
Penis of *Isoperla claudiae* sp. n. **A** ventral view of the extruded penis **B** medial penial armature, scale bar 50 µm **C** medial penial armature, scale bar 50 µm **D** scales of the medial penial armature, scale bar 20 µm **E** scales found caudally the medial penial armature, scale bar 20 µm **F** medial penial armature of *Isoperla orobica*, scale bar 200 µm. Photographs **A–E** by W. Lechthaler, Vienna.

Penis (Fig. 2): Divided into four lobes and a basal section in extruded position. Medial penial armature located on the medial lobe adjacent to the ventral lobe, lateral penial armatures lacking. The medial penial armature resembles an equilateral triangle of 130 μm width and 97 μm length formed by slightly brownish coloured scales that are relatively blunt and short and vary in length (4.98–6.26 μm). The median basal area is sparsely covered by shorter scales. The medial penial armatures are connected distally by a narrow band of colourless scales with an area densely covered by smaller triangular scales. Similar scales are located proximal to the medial armatures. Their length varies between 4.4 and 7.2 μm. With the exception of the medial armatures the central area of the ventral penis is bald. Lateral portions of lateral lobes covered by dense scales similar to the ones on medial and ventral lobes, being denser at the connection to the ventral lobe.

Female abdomen (Fig. 1D): 1^st^ to 7^th^ tergite dorsally brown with increasing laterally whitish areas towards the apex, 8^th^ to 10^th^ tergite mostly yellowish with small brown medial patches. Laterally and ventrally all segments entirely whitish with dark markings reduced to delicate brownish lines at the posterior end of sternites. Subgenital fig covers most of sternite VIII width and half of sternite IX length, posterior margin rounded semicircularly. Sternite X and paraprocts yellowish; cerci generally brownish, the first segment being pale.

**Figure 3.**
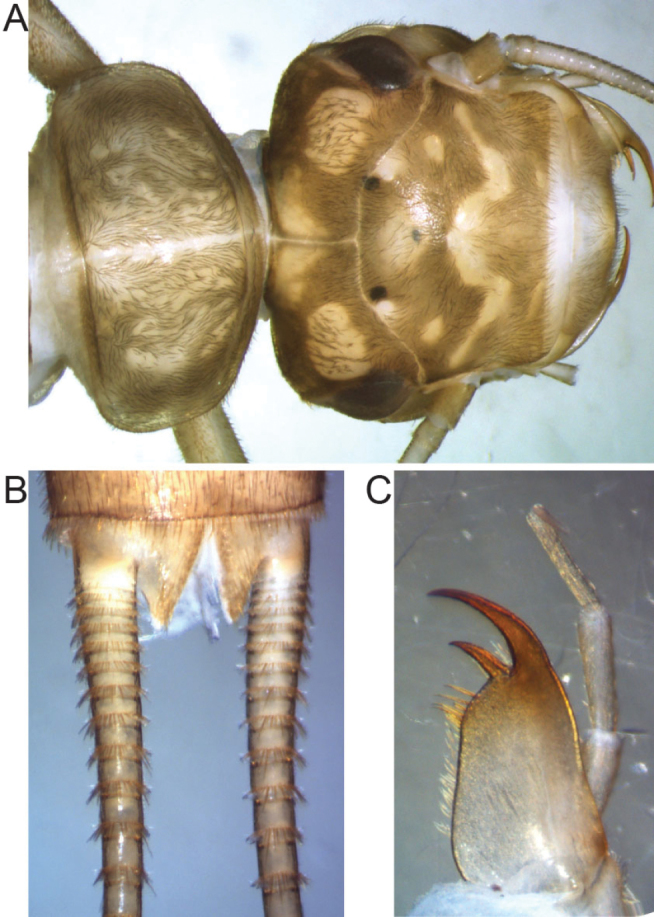
Larval characters of *Isoperla claudiae* sp. n. **A** mature larva of *Isoperla claudiae* sp. n. in dorsal view, head and pronotum **B** ventral view of the abdomen end of *Isoperla claudiae* sp. n. **C** lacinia of *Isoperla claudiae* sp. n.

Larva (Fig. 3): Body length of the matured larva: 13 mm. General colour brown but with pale markings. Pilosity dense, pronotal, posterior tergal and cercal fringes short and acute; swimming hairs lacking. Head dark brown with two yellow spots anterior to the M-line, two posterior to the M-line, one around the median occellus and one laterally to the each posterior ocellus. Two large pale spots laterally on the occipit (Fig. 3A). M-line distinct, tentorial callosities hardly visible; eyes normal sized. Scape and pedicel pale, the following antennomeres light brown; palpi yellowish, mouthparts light brown. Lacinia triangular, with 6 strong setae beneath the two apical teeth, thin hairs present all along the inner margin; galea with scattered setae on the whole surface (Fig. 3C). Pronotum rectangular with rounded corners, twice as wide as long, brown but with a narrow medial pale stripe along the medial suture and a marbled impression due to several medial pale areas, lateral parts uniformly brown, margins laterally pale. Mesonotum and metanotum mostly brown but with a pale, marmoreal pattern; wingpads brownish. Ventral surface of thorax pale, furcasternites and furcal pits inconspicuous. Legs uniformly pale. Abdominal tergites brown with a pair of roundish pale spots laterally to a median, darker area. The spots are increasing in size towards the entirely pale last tergite. Ventral surface of abdomen pale brown, the distal segments darker. Paraprocts brown; cerci light brown with dense circumferential rows of bristles of varying length at the end of each segment.

#### Ecology and distribution.

The species was collected in a small spring-brook at 535 m a.s.l. in the Karawanken, and a small forest brook at 980 m a.s.l. in the Kamnik Alps (Southern Limestone Alps).

#### Preliminary description of the male drumming call.

Since only one signal from a single male could be recorded we cannot give any information on the variation range of the signal parameters in this species. The aim of this preliminary description is only to report the basic features of the signal, but even that should be treated with some caution since we cannot be sure whether or not the recorded signal shows some deviant features.

**Figure 4.**
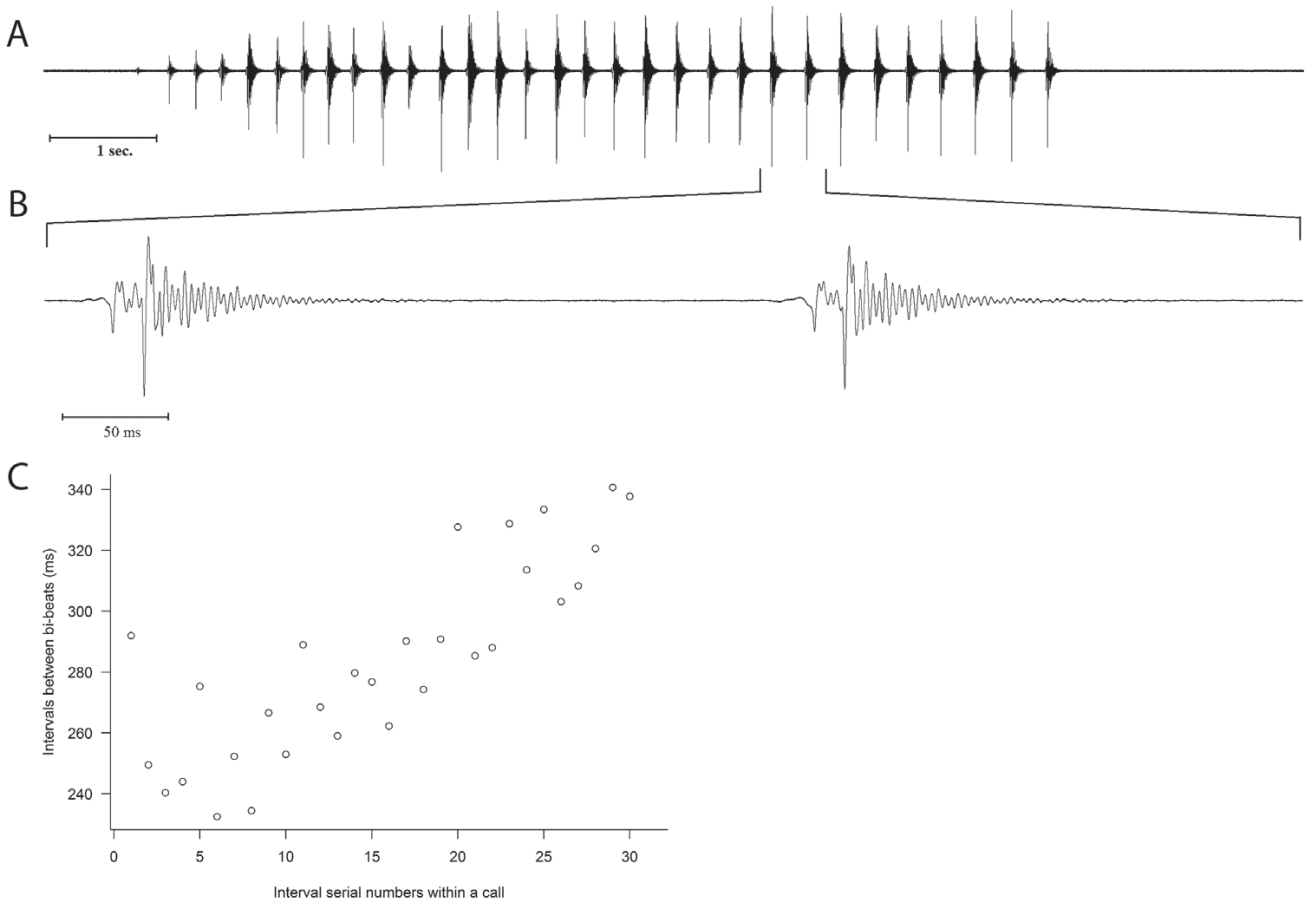
Oscillograms showing the drumming call of an *Isoperla claudiae* sp. n. male (ambient air temperature 24.2 °C). **A** oscillogram showing rhythm and amplitude variation patterns of a call **B** a faster oscillogram of two bi-beats from the second half of the call **C** variation of interval duration between bi-beats during the call presented in **A.** Inter-beat intervals were measured from the amplitude peak of one bi-beat to the amplitude peak of the next bi-beat (measured on the same call presented in **A**).

As it is observable in (Fig. 4A) the male call is a sequence of bi-beats. After an initial crescendo the peak amplitude of bi-beats fluctuate around a constant value. In bi-beats the first beat is of lower amplitude (missing in the low amplitude initial part of the call and sometimes hardly detectable even in the main part of the signal), the second one is of higher amplitude and followed by a long, decaying wave train (Fig. 4B). Inter beat interval within bi-beats varied between 8–20 ms during the call. The interval between bi-beats (or single beats at the initial part) gradually increased during the sequence (except for a short initial part of the sequence where inter beat interval decreases) varying between 230–350 ms (Fig. 4C).

## Discussion

### Relationships

The new species can be attributed to the *oxylepis* species-group sensu Murányi (2011), currently comprising *Isoperla oxylepis oxylepis* (Despax), *Isoperla oxylepis balcanica* Raušer, *Isoperla bosnica* Aubert, *Isoperla orobica* Ravizza and *Isoperla submontana* Raušer. These species develop similarly shaped medial penial armatures and scales of penial armatures, lack real lateral armatures, and develop dense, uncoloured scales on each lobe.

*Isoperla claudiae* sp. n. is most similar to *Isoperla orobica*, a species restricted to the south-western Alps, but can be easily distinguished from the latter species as the scales of the medial penial armature are shorter in *Isoperla claudiae* sp. n., a higher density of uncoloured scales on the penis in *Isoperla claudiae* sp. n., as well as yellow, hardly visible rugosities of the pronotum in *Isoperla claudiae* sp. n.

The male drumming call of *Isoperla claudiae* sp. n. is clearly different from the drumming call of *Isoperla oxylepis*, which is the only species of the *Isoperla oxylepis* species-group, where published information regarding the vibratory signals is available (Rupprecht 1969, 1983). Amongst the European species of *Isoperla* the male call of *Isoperla claudiae* sp. n. is most similar to that of *Isoperla rivulorum* (Pictet) (Rupprecht 1969, Tierno de Figueroa and Sánchez-Ortega 1999, Tierno de Figueroa et al. 2000, 2002, 2011, Tierno de Figueroa and Luzón-Ortega 2002, Luzón-Ortega et al. 2010), but the beat group repetition period seems to be longer in this species (230–350 ms, 24.2 °C, Fig. 4C) than in *Isoperla rivulorum* (Luzón-Ortega et al. 2010) reported 103–163 ms at 20 °C), and *Isoperla rivulorum* frequently produces 3 beats per beat group.

### The Southern Limestone Alps as centers of endemism

The southern slopes of the Alps from the Ligurian Prealps in the southwest to the Pohorje Mountains in the east are densely covered by microendemic species. Concentrations of endemic species in the south and south-eastern Alps are well known among Trichoptera species (Malicky 1983, 2000); regarding Plecoptera *Leuctra dylani* Graf, *Leuctra juliettae* Vinçon & Graf, *Leuctra muranyii* Vinçon & Graf and *Protonemura bipartita* Consiglio are restricted to small areas from the Bergamo prealps to the Lessinian Alps; the apterous *Leuctra istenicae* Sivec and *Siphonoperla ottomoogi* Graf are nested as microendemics in southeastern refugia referred to as the Steirische Randgebirge.

The western alpine slopes (Biellese, Graian and Cottian Alps) are another area of alpine endemism where a high diversity within the genus *Leuctra* is found (Ravizza Dematteis and Ravizza 1988; Vinçon and Ravizza 1998), and their distribution patterns are associated with the presence of nunataks during the Würm glaciation (Ravizza and Ravizza Dematteis 1993, 1994). The hitherto known range of *Isoperla claudiae* sp. n. fits well in this hot-spot of biodiversity and supports the Dinodal theory of Malicky (1983, 2000), which suggests glacial species-specific refugia within the Alps based on distribution patterns of endemic caddisfly species. Most of these microendemic species are stenoecious elements of springs and small streams in medium altitudes.

## Supplementary Material

XML Treatment for Isoperla claudiae
